# Mature adipocytes observed to undergo reproliferation and polyploidy

**DOI:** 10.1002/2211-5463.12207

**Published:** 2017-03-09

**Authors:** Pengfei Xu, Jiao Li, Jin Liu, Jing Wang, Zekai Wu, Xiaotian Zhang, Yonggong Zhai

**Affiliations:** ^1^ Beijing Key Laboratory of Gene Resource and Molecular DevelopmentCollege of Life SciencesBeijing Normal UniversityChina; ^2^ Department of Biology Science and TechnologyBaotou Teacher's CollegeChina; ^3^ Key Laboratory for Cell Proliferation and Regulation Biology of State Education MinistryInstitute of Cell BiologyCollege of Life SciencesBeijing Normal UniversityChina

**Keywords:** adipocytes, lipid droplets, phase‐contrast, polyploidization

## Abstract

Lipid‐filled mature adipocytes are important for the study of lipid metabolism and in the development of obesity, but whether they are capable of reproliferation is still controversial. Here, we monitored lipid droplet dynamics and adipocyte reproliferation in live, differentiated 3T3‐L1 cells using a phase‐contrast microscope in real time. Phase‐contrast microscopy achieves a similar visual effect *in situ* to that obtained using traditional dyes such as Oil Red O and BODIPY *in vitro*. Using this method, we captured the process that lipid droplets use for dynamic fusion in living cells. Unexpectedly, we acquired images of the moment that differentiated 3T3‐L1 cells containing lipid droplets entered mitosis. In addition, we observed some binucleated mature adipocytes. This information provides a better understanding of the adipocyte differentiation process.

**Abbreviations**
DAPI: 4,6‐diamidino‐2‐phenylindole
FBS: fetal bovine serum
MCE: mitotic clonal expansion
ORO: Oil Red O
RT‐PMO: real time phase‐contrast microscope observation

Lipid droplets, also known as lipid bodies, oil bodies and adiposomes, are important dynamic cellular organelles that are used for storage of neutral lipids 1. Almost all bacterial and eukaryotic cells have the ability to accumulate neutral lipids and maintain them as an energy reservoir in lipid droplets. Lipid droplets generally have a spherical shape; they consist of a neutral lipid core (mainly of triglycerides and cholesteryl esters) surrounded by a phospholipid monolayer surface decorated with integral and peripheral proteins 2, 3. Initially, lipid droplets were considered to be merely an inert depot of excess lipids within cells. Recent discoveries, however, have revealed that they are actively engaged in a wide range of metabolic disorders such as obesity, diabetes, steatosis, atherosclerosis, inflammation and cancer 4, 5, 6. However, the exact mechanisms underlying the diverse functions of lipid droplets are still far from clear. The study of lipid droplets has attracted much attention from cell biologists. 3T3‐L1 is a classic cell line originally developed by clonal proliferation from Swiss mouse embryo tissue. Owing to its potential for differentiation from a fibroblastic phenotype into an adipocyte with lipid droplets, this cell lineage is widely used as an *in vitro* model for adipogenesis and lipid droplet formation 7. Whether lipid‐filled mature adipocytes are capable of reproliferation is still controversial and there has been no study on polyploidization in mature adipocytes.

Polyploidization, alternatively called whole‐genome amplification, refers to eukaryotic organisms containing more than two homologous basic sets of chromosomes. Polyploidy was first found in plants more than a hundred years ago, and it is especially common in angiosperms. In mammals, polyploid cells can occur during some physiological processes, such as some tissue development (liver, skeletal muscle, heart, placenta, brain and bone marrow) 8, and also during pathological processes, such as hyperthyroidism (thyroid cells) 9, hypertension (vascular smooth muscle cells) 10 and tumorigenesis (esophageal and colorectal cancers 11, lung and bronchus cancers 12, breast cancers 13, prostate cancers 14, kidney and renal pelvis carcinoma 15, bladder cancer 16, thyroid cancer 17, some types of leukemia 18, glioblastoma 19 and melanoma, and rare childhood tumors 20).

In this study, we monitored lipid droplet dynamics in 3T3‐L1 and mouse primary adipocyte over a long time course using a live cell imaging system with phase contrast microscopy. We refer to this method as real time phase‐contrast microscope observation (RT‐PMO). Compared with staining with Oil Red O (ORO) and BODIPY, RT‐PMO can achieve a similar observation during the differentiation of pre‐adipocytes *in situ*. Furthermore, we captured the real time moments when lipid droplets underwent dynamic fusion in living cells and when differentiated 3T3‐L1 cells containing lipid droplets divided into two daughter cells. We also observed some polyploids in the mature adipocytes. Overall, our findings will help to better understand the adipocyte differentiation process and the development of obesity.

## Materials and methods

### Cell isolation and cell culture

Mouse primary adipocytes were isolated and cultured from gonadal fat pads in 4‐week‐old C57BL/6 mice, as previously described 21. All procedures were performed in accordance with the guidelines and regulations of the Ethics and Animal Welfare Committee of Beijing Normal University. Briefly, the white adipose tissue pieces were minced in Dulbecco's modified Eagle's medium (DMEM) on ice and were transferred to a digestion buffer with 0.2% (w/v) collagenase (type I, Sigma‐Aldrich, St. Louis, MO, USA) in DMEM containing 0.1% (w/v) bull serum albumin. The digestion was performed for 30 min at 37 °C with continuous shaking (120 r.p.m.). We placed the cell strainer (70 μm) into a funnel and filter‐digested the tissue into a 50 mL conical tube. The cell suspension was allowed to settle for 15 min on ice and was then centrifuged at 500 ***g*** for 10 min. Most of the supernatant was discarded, and the precipitate was washed three times by centrifugation in PBS at 4 °C. The pellet was resuspended in culture medium, and the cells were counted and seeded for culture. The culture medium was DMEM with 10% fetal bovine serum (FBS), 1% penicillin–streptomycin and 5 ng·mL^−1^ epidermal growth factor (Invitrogen/Thermo Fisher Scientific, Waltham, MA, USA) at 37 °C, with 5% CO_2_ 22. The murine 3T3‐L1 pre‐adipocyte cell line was obtained from the Cell Resource Center, Peking Union Medical College (Beijing, China), and was cultured in DMEM containing 10% newborn calf serum and 1% penicillin–streptomycin. To induce pre‐adipocyte cell differentiation, 2 days after reaching confluence, the cells were cultured in differentiation medium, which was DMEM containing 10% FBS, 10 μg·mL^−1^ insulin (Sigma‐Aldrich), 0.25 μm dexamethasone (Sigma) and 0.5 mm methylisobutylxanthine (Sigma‐Aldrich). Cells were then incubated in DMEM supplemented with 10% FBS and 10 μg·mL^−1^ of insulin for another 2 days, followed by DMEM including 10% FBS with medium changes every 2 days for an additional 4 days or more.

### Antibodies and histochemical staining

Rabbit anti‐perilipin antibody diluted at 1: 250 was purchased from Cell Signaling Technology (Danvers, MA, USA). Secondary antibody staining was accomplished using Alexa Fluor 488 goat anti‐rabbit IgG following the manufacturer's recommendations. BODIPY 493/503 (Invitrogen/Thermo Fisher Scientific) and 4,6‐diamidino‐2‐phenylindole (DAPI, Sigma‐Aldrich) were added to the fixed cells for 5 min at room temperature.

The protocol for ORO staining was derived from that developed by Koopman with minor modifications 23. ORO (Sigma‐Aldrich) dissolved in isopropanol (0.5%) was incubated for 1 h at 60 °C, filtered, mixed with distilled water (3: 2), and finally filtered twice before use. The cells were washed three times with PBS and fixed with 3.7% paraformaldehyde for 20 min. Then, 0.2% ORO was added to the fixed cells for 20 min at 60 °C, and then washed with 60% isopropanol. If desired, the cells were counterstained using Mayer's hematoxylin for 60 s to visualize the nuclei.

### Phase‐contrast and immunofluorescence microscopy images and movies

Real time phase‐contrast microscope observation images and *in situ* images of ORO‐ or BODIPY‐stained cells were taken using an Axio Observer Z1 (Carl Zeiss, Jena, Germany) equipped with an EC Plan‐Neofluar ×10/0.30 ph1 phase contrast objective and a centered condenser phase stop. Phase‐contrast brightfield and BODIPY‐stained images were acquired using an Axio CamMR3 monochrome camera; ORO images were simultaneously obtained using an Axio CamMR5 color camera at a corresponding position. RT‐PMO movies were captured using a live cell imaging system (at 37 °C, with 5% CO_2_) based on the Axio Observer Z1 equipped with an A‐Plan ×10/0.25 ph1 phase contrast objective and an Axio CamMR3 camera. A lipid droplet dynamic fusion movie (Movie S1, see Supporting Information) was captured every 5 s per image for 20 min; and a living differentiated 3T3‐L1 cell with lipid droplets division movie (Movie S2) was captured every 2 min per image for 19 h. The contrast and brightness of the images and movies acquired with the Axio Observer Z1 were regulated using Axio Vision LE. Immunofluorescence imaging of perilipin‐stained cells was performed using a confocal laser scanning microscope LSM700 (Carl Zeiss) with a Plan‐Apochromat ×63/1.40 Oil DIC M27 objective. The filter set was for Alexa Fluor 488 and DAPI. The contrast of the images was adjusted using the ZEN 2009 light edition. The figures and movies were confirmed in more than three separate repeated trials.

## Results and Discussion

**Figure 1:**
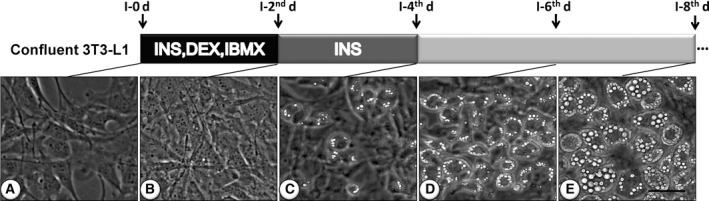
Diagram of the differentiation protocol for 3T3‐L1 differentiation and RT‐PMO of lipid droplets at different differentiation stages. (A) Induction day 0, (B) second day of induction, (C) fourth day of induction, (D) sixth day of induction, and (E) eighth day of induction. Bar: 50 μm.

A phase contrast microscope is commonly found in biological laboratories, especially in those participating in studies of transparent and colorless specimens. Lipid droplets have a high density of triglycerides and cholesteryl esters 24, and a phase‐contrast microscope exploits differences in the refractive index of different materials to distinguish lipid droplets from other cellular structures. In the present study, we applied an RT‐PMO technique to record the images of lipid droplets in live differentiated 3T3‐L1 cells that were known to include numerous large lipid droplets. As shown in Fig. 1, the lipid droplets became increasingly clear with increasing induction times. After 8 days of induction, the technique easily distinguished the lipid droplets (Fig. 1E). Furthermore, we captured dynamic fusion of lipid droplets in living 3T3‐L1 cells in real time (Movie S1).

**Figure 2:**
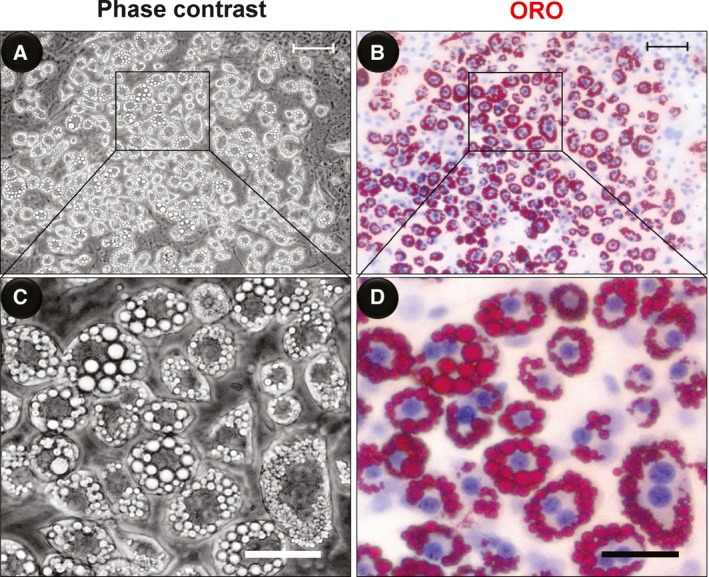
*In situ* image of phase‐contrast (A, C) and ORO‐stained (B, D) differentiated 3T3‐L1 cells. Bars: 100 μm (A, B), 50 μm (C, D).

Traditionally, ORO is a classical lysochrome diazo dye that has been extensively used for the staining of lipid droplets 23. In this method, cells should be fixed before being dyed because the fixing and staining procedures may disturb the structure of the lipid droplets in adipocytes 25. However, because unstained lipid droplets cannot be observed in live adipocytes, we do not know whether the fixing process brings allogenic material into the lipid droplet structures, and we cannot see dynamic changes in the lipid droplets in living cells. We compared the microscope images of RT‐PMO and ORO‐stained differentiated 3T3‐L1 cells *in situ*. Figure 2 shows that phase‐contrast imaging is comparably effective to ORO staining in the observation of lipid droplets.

**Figure 3:**
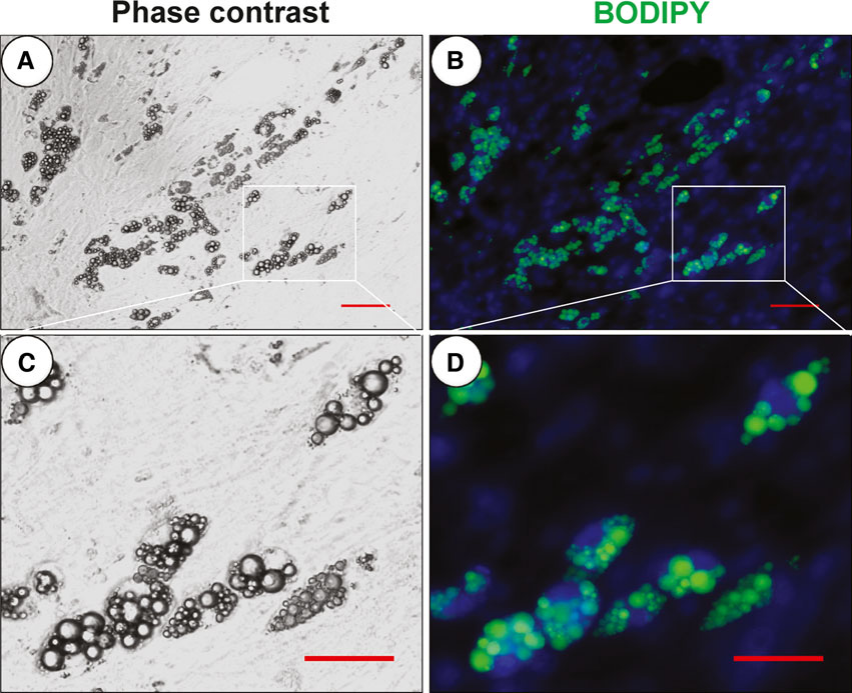
Phase‐contrast (A, C) and BODIPY‐stained (B, D) fixed mouse primary adipocyte *in situ* images. Bars: 100 μm (A, B), 50 μm (C, D).

BODIPY, a class of lipophilic fluorescent dyes, emits bright green fluorescence and has been frequently used to label lipid droplets, and has thus been convenient for double fluorescence labeling in adipocytes 26. BODIPY can stain live cells without the need for fixing, but is toxic to living cells. In addition, its fluorescence dims with cell growth. As shown in Fig. 3, RT‐PMO imaging gave similar results to BODIPY‐labeled lipid droplets in mouse primary adipocytes.

Among the various models used for studying the process of pre‐adipocytes differentiating into adipocytes *in vitro*, the 3T3‐L1 cell line is very important. It has allowed us to gain a detailed perspective on adipocyte proliferation, differentiation, transcriptional activation and the repression of lipogenesis and other aspects of adipocyte biology, which have been described in some excellent reviews 27, 28, 29, 30, 31, 32. Confluent 3T3‐L1 pre‐adipocytes can be differentiated with an adipogenic cocktail consisting of methylisobutylxanthine, dexamethasone and insulin in 10% FBS. When induced to differentiate, the first growth‐arrested stage is achieved by contact inhibition after pre‐adipocytes have been cultured to confluence 28. Upon hormonal cocktail induction, growth‐arrested 3T3‐L1 pre‐adipocytes immediately reenter the cell cycle, trigger DNA replication and undergo at least two rounds of cell division, a phase often referred to as mitotic clonal expansion (MCE) 27, 29, 33. MCE has been considered necessary for the subsequent differentiation processes and the expression of specific adipogenic transcription factors, as well as cell cycle regulators, during this period. MCE is followed by cell‐cycle arrest 28, 34. The differentiated 3T3‐L1 cells then exit the cell cycle, change their fibroblastic morphology, accumulate lipid droplets and take on the appearance of mature adipocytes 27. Generally, most researchers think that the mature adipocytes cannot undergo mitosis after they have accumulated lipid droplets. Fortunately, we captured the moment that differentiated 3T3‐L1 adipocytes containing lipid droplets were dividing by using a live cell imaging system under RT‐PMO (Movie S2). These results showed that mature adipocytes still have the ability to undergo cell division. This knowledge will help us to better understand the adipocyte differentiation process.

**Figure 4:**
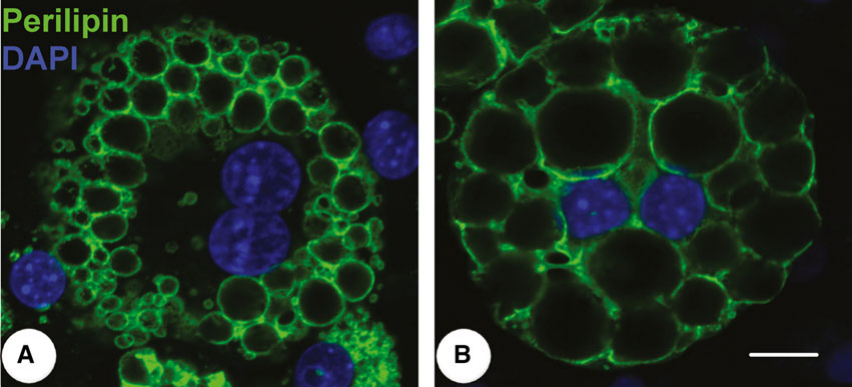
Representative binucleated cells with double labeling of perilipin (green) and DAPI (blue) in induced differentiated 3T3‐L1 cells at the 10th day (A) and the 20th day (B). Bar: 10 μm.

Furthermore, we found that the induced differentiated 3T3‐L1 cells have a double‐nucleus appearance on day 10 (Fig. 4A) and day 20 (Fig. 4A, Movie S3); the binucleated mature adipocytes are also shown in Figs 2 and 3. In previous studies, there is some evidence of mature adipocytes with a double‐nucleus appearance, which are polyploid adipocytes (Nan *et al*., fig. 6A 35; Verstraeten *et al*., figs 1G, 2A (15,16) and 4A(3,5) 36; Kuerschner *et al*., figs 7A and 9 37; Bochet *et al*., fig. 4E 38), even though the authors did not characterize or note these phenomena in the papers. In our opinion, the formation of polyploid adipocytes may be due to lipid droplets disrupting the normal cytoskeleton and causing the failure of cytokinesis. That mature adipocytes have two or multiple nuclei during the differentiation process has not been adequately described, and the exact mechanism of this process needs further research. Such studies will help us to clarify the ‘rules’ of lipid droplet accumulation in adipocytes. Moreover, whether mature adipocytes can divide or form polyploids *in vivo* requires further confirmation. This may be a biological event that is stimulated *in vivo* and may explain why obese people become overweight more quickly in special cases such as when using hormones or when they have endocrine disorders. Our findings therefore provide a novel perspective on obesity development.

## Author contributions

YZ, PX and XZ conceived and designed the project, PX, JL, JL and ZW performed the experiments, PX, JL and JW analyzed the data, PX wrote the manuscript, and YZ and XZ determined the final content of the manuscript.

## Supporting information


**Movie S1**. RT‐PMO‐monitored lipid droplet dynamic fusion in the differentiation process of live 3T3‐L1 cells.

**Movie S2**. RT‐PMO‐monitored reproliferation of mature adipocytes in live differentiated 3T3‐L1 cells.

**Movie S3.** 3D stack of anti‐perilipin stained adipocyte at 20th day of induction.
